# Case Report: Severe palmoplantar hyperkeratosis and pain four months post-tepotinib initiation: A hand-foot skin reaction

**DOI:** 10.1016/j.jdcr.2025.05.056

**Published:** 2025-08-27

**Authors:** Catherine Laferté, Alexandre Lemieux

**Affiliations:** a Division of Dermatology, Department of Medicine, CHUM, Université de Montréal, Montréal, Quebec, Canada; b Division of Dermatology, Department of Medicine, Hôpital du Sacré-Coeur-de-Montréal, Montréal, Quebec, Canada

**Keywords:** Acral Hyperkeratosis, Dermatological Toxicities, drug reaction, Hand-Foot Reaction, Lung Adenocarcinoma, Pain, Palmoplantar Keratoderma, skin of color, Tepotinib, Topical Corticosteroids

## Introduction

Chemotherapy-related dermatologic adverse events are a well-recognized complication in oncology. These cutaneous toxicities are prevalent with certain chemotherapeutic agents and targeted therapies.^1^

Hand-foot syndrome (HFS) typically presents with erythema, edema, and pain localized to the palms and soles, and is commonly associated with capecitabine, 5-fluorouracil, and liposomal doxorubicin.^1^^,^^2^ In contrast, hand-foot skin reaction (HFSR) presents with distinctive hyperkeratotic plaques on areas subjected to friction.^1^ Drugs such as multikinase and vascular endothelial growth factor inhibitors are commonly implicated in HFSR.^3^^,^^4^

Tepotinib, a mesenchymal-epithelial transition (MET) factor inhibitor used for advanced non-small cell lung cancer with MET exon 14 skipping mutations, has limited documentation regarding its dermatologic adverse effects. Prior reports have primarily focused on other cutaneous reactions to MET inhibitors, such as papulopustular and acneiform eruptions, and pruritus^5^, ^6^, ^7^ leaving acral hyperkeratosis underreported with 2 cases of HFSR published to date.^8^^,^^9^

## Case report

An 83-year-old Haitian woman was referred in June 2024 for severe palmoplantar keratoderma and pain since May 2024. She had stage 4 lung adenocarcinoma (PD-L1 100%) with a cMet exon 14 skipping mutation and had been on oral tepotinib since June 2023 for palliative care. Her history included cervical squamous cell carcinoma, arterial thrombosis post-cisplatin, COPD, and active smoking.

**Fig 1:**
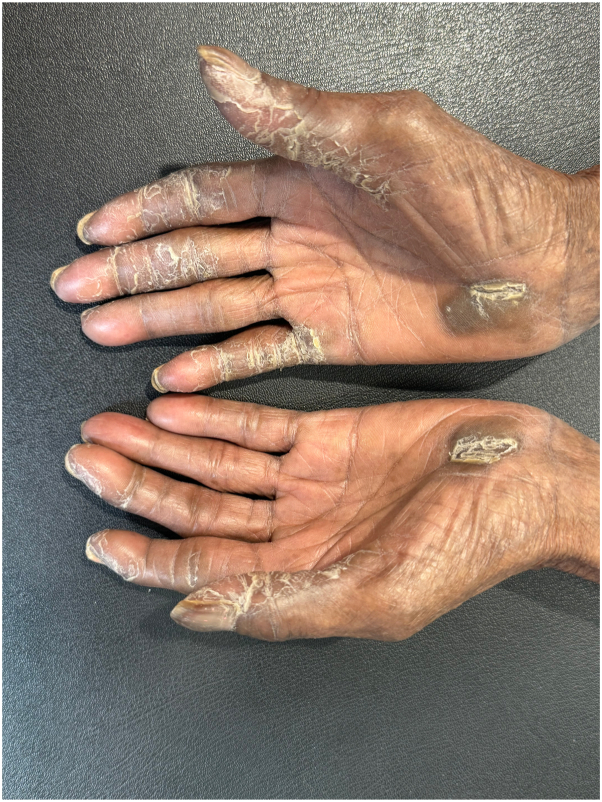
Palmar hands at initial presentation.

**Fig 2:**
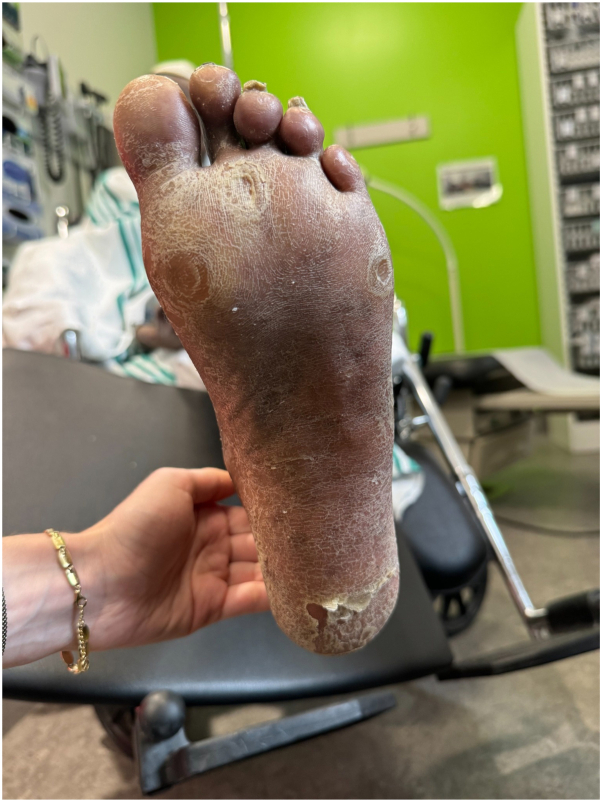
Plantar surface of the foot at initial presentation.

The patient initially developed acral hyperkeratosis on her right palm, which progressed to involve both hands and feet within 4 months after initiation of tepotinib. By spring 2024, the condition had become severely painful, limiting hand function. Dermatologic examination revealed thick, non-friable hyperkeratotic plaques on the palmar aspects of the fingers and palmar eminences, along with hyperpigmentation, which was more prominent due to darker skin phototype. Significant subungual hyperkeratosis, minimal nail dystrophy, mild clubbing, and pulp pain in the fingers was noted. The feet were notable for plantar hyperkeratosis and callosity formation [Figs 1 and 2]. Mucosa, scalp, elbows, and knees were unremarkable. No erythroderma was present.

Culture of nail debris was positive for *Candida orthopsilosis* 2+ (mild growth), which did not appear to correlate with the clinical presentation.

Symptomatic treatment was initiated with a compounded preparation of 10% salicylic acid in a petroleum base, along with betamethasone dipropionate 0.05%, applied twice daily to the hands and feet for 1 month, followed by as-needed application. The patient improved slightly with the topical treatments, but tepotinib was discontinued due to severe pain, allowing a drug holiday per the oncology team. A topical vasodilator (nitroglycerin) was not trialed, although vasodilators have been hypothesized to provide relief in VEGFR-HFSR cases. Further studies are needed to determine their role in MET inhibitor-induced HFSR.^10^

**Fig 3:**
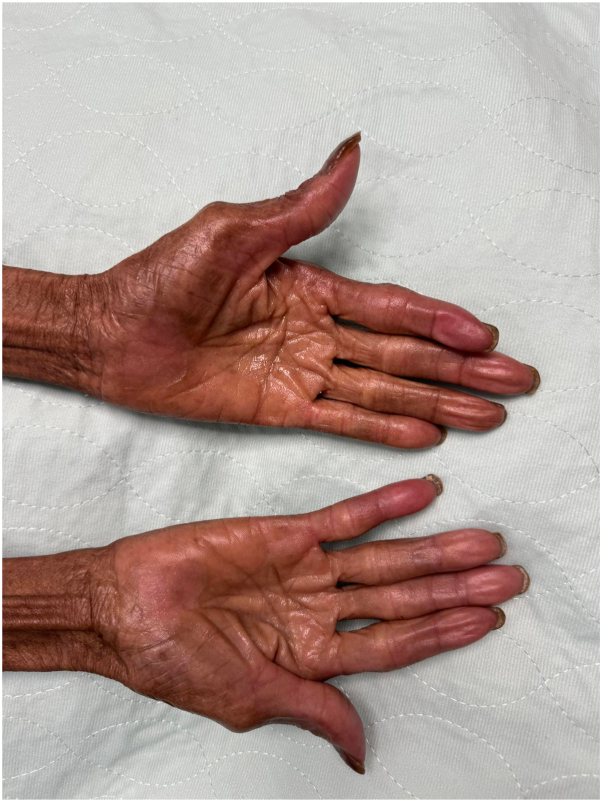
Palmar hands, 1 month posttreatment.

One month after tepotinib cessation and topical treatment initiation, symptoms fully resolved in both hands and feet [Fig 3]. In July 2024, immunotherapy was resumed at a reduced dose of 225 mg PO daily, which is half the usual dosage, following a 2-month suspension. Topical therapy was continued preventively. After 1 month on the reduced dosage, mild hand desquamation recurred but was well tolerated. Meanwhile, metastatic disease showed sustained radiologic response. The topical regimen was maintained, allowing continued immunotherapy.

## Discussion

Dermatologic adverse events such as HFS and HFSR are significant complications of cancer treatments, often necessitating dose modifications or interruptions.

Multiple overlapping terms exist in the literature for toxic effects of chemotherapy (TEC), including palmoplantar erythrodysesthesia, eccrine squamous syringometaplasia, and intertrigo eruption. These are encompassed under the broader category of toxic erythema of chemotherapy. When affecting the palms and soles, TEC is often referred to as HFS, particularly in cases of diffuse glazed erythema and mild hyperkeratosis due to capecitabine.^1^ HFS is commonly associated with cytotoxic agents such as capecitabine, 5-fluorouracil (5-FU), and liposomal doxorubicin, and presents with diffuse erythema, swelling, and dysesthesia. It is attributed to direct toxicity to epidermal basal cells. Capecitabine-induced HFS often occurs within the first 2 to 6 weeks of treatment.^11^ For 5-FU, symptoms may develop within days to weeks after therapy initiation.^11^ Liposomal doxorubicin-induced HFS typically appears after 2 to 3 cycles of treatment.^12^

HFSR, on the other hand, is primarily associated with multikinase inhibitors, including sorafenib, sunitinib, regorafenib, and cabozantinib. It presents with localized, well-demarcated hyperkeratosis in friction-prone areas due to impaired tissue repair caused by kinase inhibition, typically developing within the first 2 to 4 weeks of treatment. These medications target multiple kinases involved in tumor growth, angiogenesis, and metastasis, particularly vascular endothelial growth factor receptor (VEGFR), platelet-derived growth factor receptor, and c-KIT. Given the patient’s presentation and response to dose modification, the term HFSR is more appropriate for tepotinib-induced acral hyperkeratosis. A paraneoplastic phenomenon was considered due to the delayed rash onset but deemed less likely, given the improvement with lower tepotinib doses.

Previous authors have suggested that the mechanism underlying VEGFR inhibitor-induced HFSR involves endothelial dysfunction and impaired microvascular repair, leading to friction-induced hyperkeratosis and pain. While the exact pathophysiology of MET inhibitor-induced HFSR remains unclear, overlapping mechanisms with VEGFR inhibition may explain this case.

VEGFR inhibitors impair vascular repair in mechanically stressed areas, causing localized microvascular damage and hyperkeratosis. In contrast, MET signaling regulates keratinocyte proliferation and tissue regeneration. MET inhibition disrupts these processes, delaying wound healing and leading to acral hyperkeratosis similar to VEGFR inhibitor-induced HFSR, but with a slower progression.^4^^,^^13^^,^^14^ MET and VEGFR pathways also interact in tissue repair. MET activation downregulates VEGFR2, impairing vascular repair, while VEGFR inhibition may induce compensatory MET activation to support keratinocyte survival. Dual inhibition disrupts this response, resulting in chronic hyperkeratosis in friction-prone areas.^15^^,^^16^

Notably, VEGFR inhibitor-induced HFSR appears early (within 2-4 weeks), whereas MET inhibitor-induced HFSR, as seen in this case, presents several months later, likely due to progressive keratinocyte dysfunction rather than immediate endothelial damage.^17^

Tepotinib-induced palmoplantar hyperkeratosis differs from typical hand-foot reactions, with a delayed onset of 4 months in this case and severe progression by 1 year. This highlights the need for prolonged monitoring of MET inhibitor-related skin toxicities. Clinicians should consider dose reduction or discontinuation alongside topical management to control symptoms while maintaining oncologic treatment. As MET inhibition primarily affects keratinocyte function rather than vascular integrity, the role of vasodilators remains uncertain. Further research is needed to clarify mechanistic differences between MET and VEGFR inhibitor-induced HFSRs and to optimize management strategies for improved patient outcomes and quality of life.

## Conflicts of interest

None disclosed.
